# Technical Advances in Single-Cell RNA Sequencing and Applications in Normal and Malignant Hematopoiesis

**DOI:** 10.3389/fonc.2018.00582

**Published:** 2018-12-07

**Authors:** Xiang-tao Huang, Xi Li, Pei-zhong Qin, Yao Zhu, Shuang-nian Xu, Jie-ping Chen

**Affiliations:** Center of Haematology, Southwest Hospital, Third Military Medical University (Army Medical University), Chongqing, China

**Keywords:** single-cell RNA sequencing, normal hematopoiesis, malignant hematopoiesis, hematopoietic hierarchy, hematological malignancy

## Abstract

Single-cell RNA sequencing (scRNA-seq) has been tremendously developed in the past decade owing to overcoming challenges associated with isolation of massive quantities of single cells. Previously, cell heterogeneity and low quantities of available biological material posed significant difficulties to scRNA-seq. Cell-to-cell variation and heterogeneity are fundamental and intrinsic characteristics of normal and malignant hematopoietic cells; this heterogeneity has often been ignored in omics studies. The application of scRNA-seq has profoundly changed our comprehension of many biological phenomena, including organ development and carcinogenesis. Hematopoiesis, is actually a maturation process for more than ten distinct blood and immune cells, and is thought to be critically involved in hematological homeostasis and in sustaining the physiological functions. However, aberrant hematopoiesis directly leads to hematological malignancy, and a deeper understanding of malignant hematopoiesis will provide deeper insights into diagnosis and prognosis for patients with hematological malignancies. Here, we aim to review the recent technical progress and future prospects for scRNA-seq, as applied in physiological and malignant hematopoiesis, in efforts to further understand the hematopoietic hierarchy and to illuminate personalized therapy and precision medicine approaches used in the clinical treatment of hematological malignancies.

## Introduction

Single-cell-based technologies are being rapidly developed and applied in the field of pluripotent and tissue-specific normal and malignant hematopoiesis. With recent advances in single-cell isolation platforms, including the ICELL8™ single-cell system, the Fluidigm C1 single-cell automatic preparation system and the Illumina Bio-Rad single-cell sequencing solution, it is possible to isolate single cells from multiple specimens simultaneously with high throughput and high resolution. Single-cell sequencing (SCS) has rapidly advanced along with developments in genome/transcriptome amplification and next-generation sequencing (NGS). The term SCS consists of single-cell genome sequencing, single-cell transcriptome sequencing, namely single-cell RNA sequencing (scRNA-seq), and single-cell epigenome sequencing (1, 2). There exists remarkable heterogeneity and functional distinction among homogenous cell populations, owing to diverse epigenetic states, including DNA methylation, hydroxymethylation, histone modification, chromatin configuration, noncoding RNA regulation, and structural and regulatory proteins bound to chromatin. Currently, single-cell epigenome sequencing refers primarily to single-cell DNA methylation sequencing and single-cell assay for transposase-accessible chromatin using sequencing (Single-cell ATAC-seq).

Generally, most interpretations of basic experimental and clinical results *in vivo* or *in vitro* are based on the assumption that all of the cells used are homogeneous. However, this averaging of messages processing method always loses the critical information owing to ignoring the cell-to-cell variation, even within genetically homogenous cell populations, and natural heterogeneity within specimens is not truly reflected. Cellular heterogeneity is present in nearly all organs, tissues, and tumors in multicellular organisms, and presents a challenge to discern cellular functions and roles in normal functioning and in disease states as well. Single-cell transcriptome sequencing, also known as scRNA-seq, can elucidate the composition of heterogeneous cell populations, including the cellular heterogeneity during normal and malignant hematopoiesis. The concept of cellular heterogeneity was first proposed in 1957 (3). Single cells are considered the smallest structural and functional unit of an organism, as each cell represents a unique unit with molecular coding across the DNA, RNA, and protein conversions (4). Thus, relevant studies, especially the omics studies, are expected to be carried out at the single cell level. Here, we aim to discuss the recent technical progress as well as the application of scRNA-seq in normal and malignant hematopoiesis, in efforts to better understand the hematopoietic hierarchy and to illuminate personalized therapy and precision medicine approaches used in the clinical treatment of hematological malignancies.

## Single-cell Isolation Approaches

Single-cell sequencing applied in transcriptome was first carried out by Tang's lab in 2009 (5). Undoubtedly, single-cell isolation is the critical step in scRNA-seq. At present, the main approaches for isolating single cells from heterogeneous tissues or cultured cells include serial limited dilution, micromanipulation, fluorescence-activated cell sorting (FACS), laser-capture microdissection (LCM), and microfluidics (6–9). New sophisticated methods with higher accuracy and specificity are also emerging, such as Raman tweezers (10, 11) and transcriptome *in vivo* analysis (12). The above approaches differ among their advantages and disadvantages, and each approach has a unique scope of application (Table 1). Here, we will summarize them briefly in the following.

**Table 1:** The currently used single-cell isolating methods with respective advantages and disadvantages.

**Single-cell isolating methods**	**Advantages**	**Disadvantages**	**References**
Serial limited dilution	Suitable for microbial specimens	Complicated operation, high false positive rate	(9)
Micromanipulation	Operability Low-cost Small quantity of cells	Low throughput Time consuming Mechanical shearing damage	(11)
Fluorescence-activated cell sorting	High accuracy High-throughput	High initial cell count Low efficiency Potential cell damage by rapid flow	(7)
Laser-capture microdissection	High efficiency High specificity Suitable for tissue specimen	Complicated operation Need staining	(6)
Microfluidics	High efficiency High throughput Homogeneous cells	Only for transcriptome Unable to sort variant cells	(7, 8)
Raman tweezers	Unculturable microorganisms	Potential contamination Potential physical misplacement	(10, 11)
Transcriptome *in vivo* analysis	Direct RNA capturing Non-invasive operation	Low throughput One tissue at one time	(12)

Serial limited dilution, a classical technology in single-cell separation, has been used by laboratories all over the world for decades. Owing to the stochastic distribution property of suspended cells, the number of cells in a highly diluted specimen can be as low as one single cell per well by multiple serial dilution into 96-well plates. Such method of seeding cells at low concentration is indeed easy to carry out with standard laboratory pipettors with a low reagent cost, but it is time consuming since the probability of achieving a single cell in an aliquot is of statistical nature (9). What's more, serial limited dilution is prone to a high false positive rate and to exclusion of cells of interest, which hinders the isolation of target cells. Hence, while this approach is appropriate for microbial samples, it is not a preferred approach for sorting single cells from complex specimens (11).

Individual cells can also be sorted by micromanipulation using simple mouth pipettes (13). As an operable and low-cost approach with visualization, micromanipulation is most routinely used in single cell isolation, including endosymbionts from termite gut (14) and crenarchaeota from soil (15). However, micromanipulation is time consuming, has low throughput, and can cause mechanical shearing of cells; what's worse, it is only applicable to cells in suspension.

Based on multiple cellular physical or optical properties, such as the relative size, granularity, and intrinsic or extrinsic fluorescence, fluorescence activated cell sorting (FACS) has been applied as a preferred approach in single-cell separation (11). The primary advantages of FACS-based sorting are the operability, low-cost, high levels of accuracy and high throughput. However, FACS system requires a large number of suspended cells as the initial material, which hinders the isolation of low-abundance cell populations. Worse still, sorted cells may be damaged by the high pressure flow, so cell viability should be taken into consideration if sorted live cells are planned to be used in downstream protocols (7).

Laser-capture microdissection (LCM) is an ideal method to capture single cells from tissue samples. LCM is based on the selective adherence of visually targeted cells and tissue fragments to the thermoplastic membrane activated by a low energy infrared laser pulse (6). LCM enables the capture of cells from intact tissues in a natural state, and the RNA integrity of the captured cells is well retained. LCM is highly efficient and specific, and is suitable for rapid separation of interested tissue specimens. Limitations of LCM include its complicated operation, and preparation of the tissue specimens by fixation or cryopreservation. Moreover, RNA contamination may occur during cell separation and tissue cutting.

Innovative microfluidic devices have opened new horizons in single-cell isolation and analysis (16). Fabricated microfluidic chips make up the core component of microfluidic devices; these chips allow for sample compartmentalization and control the management of nanoliter reactions. Together with accurate construction of low-volume chambers and tubes, microfluidics can serve as an ideal method for single cell separation (7). The advantages inherent to microfluidics are high throughput with minimal effort, cost effectiveness, and high accuracy, and microfluidics are suitable for isolating homogeneous cells and able to sort uncultivated cells from a small quantity of samples. However, the limitations are that microfluidics are only useful for transcriptome analysis and can't readily be used to separate variant cells.

Raman tweezers are a newly advanced sophisticated technology for isolating single cells. Raman microspectroscopy enables the identification of different cell types by biochemical profiling without external labeling. After cells have been identified by Raman microspectroscopy, they are subsequently trapped with a laser (11). Single yeast and bacterial cells have been successfully isolated from an artificial cell mixture according to their Raman spectra (10). Raman tweezers are suitable for unculturable microorganisms without any pre-treatment, but only physiologically distinct cells are permitted and exists potential contamination and physical misplacement.

Transcriptome *in vivo* analysis (TIVA), though not strictly a technique for single-cell isolation, is another innovative method that captures RNA from a single cell directly by light activation using TIVA tags. The TIVA capture tag enables researchers to target and isolate RNAs from living cells in their natural microenvironments without damaging surrounding cells, which preserves the cellular response to the microenvironment and, to a great extent, the location information of cells in their resident tissue. The TIVA approach permits cell-specific transcriptome capture from viable intact heterogeneous tissues with non-invasive operation. However, the low throughput and the limitation that a single tissue can be evaluated in each experiment hinder its widespread usage (12).

To sum up, we have described several rough principles for isolating single cells from heterogeneous cell populations. If the cells of interest are already in suspension with a relatively high abundance, FACS methodology will be the ideal method. Cells that are extremely rare can be obtained by micromanipulations. TIVA enables single-cell transcriptome analysis by directly capturing RNA from a single cell using TIVA tags. If the cells are to be isolated from a whole tissue, collagenase and dispase are routinely used. However, enzymatic digestion has adverse impacts on cells, such as influencing cell yield and immune cell function, and can alter gene transcription, which result in changes in the expression of cell surface molecules (17, 18). When tissues are prepared into cell suspensions, cells can be isolated with specific fluorescence tags. Further acquisition of single cells is a tricky step, and may require additional microfluidic processing. When cells disperse into suspension, they lose their position in the tissue; in order to take into consideration the environment of a single cell and its former neighbors, LCM are recommended. However, LCM requires detailed scanning of tissue sections, and careful attention must be paid to acquire cells of interest using microdissection. In a word, there are respective advantages and disadvantages among these single-cell isolation techniques, and the most suitable method should be chosen according to the experimental requirements.

## Single-cell RNA Amplification and Sequencing Methods

Generally speaking, a single mammalian cell only has ~10 pg of total RNA and less than 0.1 pg of mRNA. However, RNA-seq usually requires microgram amounts of total RNA for analysis, which equals the total content of millions of cells. Therefore, mRNA is reverse transcribed into cDNA, and amplification of the cDNA library is required prior to deep sequencing. Currently, there are three commonly used RNA amplification strategies in scRNA-seq: PCR-based amplification, linear isothermal amplification by T7-based *in vitro* transcription (IVT) and Phi29 DNA polymerase-based amplification. These strategies are reviewed below.

### PCR-Based Amplification and Sequencing

The PCR-based amplification is a nonlinear amplification process. The use of PCR-based amplification for preparing cDNA from single cells for scRNA-seq analysis was first reported in 1993 (19). This method was modified and applied to the first RNA-seq research in single cells in 2009 by Tang et al. (5). Based on the synthesis pattern of the second chain of cDNA, PCR-based amplification can be divided into homopolymer tailing (known as the Brady/Tang protocol), template-switching and random priming.

For the Brady/Tang protocol, after single-cell isolation and lysis, mRNAs are reverse transcribed into cDNAs using a polyT primer with a special anchor sequence. Afterwards, ~30 nt polyA tails are attached to the first-strand cDNA at the 3′-end through terminal deoxynucleotidyl transferase. Finally, the second-strand cDNA is synthesized using polyT primers with another anchor sequence. For template-switching, the second-strand cDNA is synthesized by the terminal transferase activity and the template-switching activity of reverse transcriptase, which derives three strategies for scRNA-seq, namely single-cell tagged reverse transcription sequencing (STRT-seq) (20), switching mechanism at the 5′-end of the RNA transcript sequencing (Smart-seq) (21) and Smart-seq2 (22). Random priming, another approach for amplification, involves semi-random primed PCR-based mRNA transcriptome amplification procedure. In this method, the second-strand cDNA synthesis is initiated from semi-random sequences of oligonucleotide primers. After amplification, the semi-random primers are immediately removed by BcivI, a type II restriction enzyme. Otherwise, what's different from other methods is that generated cDNA fragments are directly ligated to sequencing adaptor with no end-repairing (23). Importantly, compared to other strategies, the random priming covers full-length of any size of transcripts.

#### STRT-seq

STRT-seq is the first Smart-based scRNA-seq approach based on template-switching mechanism (20). In STRT, mRNA is reverse-transcribed into cDNA by a tailed oligo(dT) primer with a barcode and an upstream primer-binding sequence, and the barcode is introduced into the 5′-end of the transcripts by Moloney Murine Leukemia Virus reverse- transcriptase (MMLV RT).

The 3′-ends of mRNAs are preferentially amplified, and transcript quantification is accomplished through reads mapping to 5′-ends of mRNAs (20). The greatest advantage of this method is the highly accurate positioning of the 5′-end of transcripts, which diminishes the 3′-bias. Owing to the barcode strategy, this technique shows low cost and short time. Furthermore, as each mRNA relates to a single cDNA, so the number of reads observed should be directly proportional to the number of mRNA, thus it is believed that quantification can be achieved (24). However, STRT is unsuitable for applications seeking to detect alternatively spliced transcripts (25).

#### Smart-seq

Smart-seq is a robust and reproducible method for sequencing the transcriptomes of single mammalian cells with improved read coverage across transcripts and relative high sensitivity and quantitative accuracy. This strategy also relies on the terminal transferase and template-switching activities of MMLV RT (26). Briefly, polyadenylated RNA is reverse transcribed through tailed oligo(dT) priming by MMLV RT. Once the reverse transcription reaction reaches the 5′-end of an RNA molecule, a few non-templated nucleotides are added to the 3′-end of the cDNA by the terminal transferase activity of MMLV RT. This newly synthesized cDNA serves as a new template by the template-switching activity of MMLV RT, and then continues to transcribe, thus completing the amplification of transcriptome. Smart-seq greatly enriches transcripts with intact 5′-ends and eliminates the second-strand synthesis (21). The transcript amplification part of Smart-seq is now marketed as SMARTer Ultra Low RNA Kit for Illumina Sequencing.

#### Smart-seq2

Smart-seq2 was recently described as an updated version of Smart-seq (22). In this protocol, a relatively mild (hypotonic) lysis buffer containing free dNTPs and tailed oligo(dT) oligonucleotides [30-nt poly(dT) stretch and a 25-nt universal 5′ anchor sequence] is used to lyse cells without interfering with or inhibiting reverse transcription. Exchanging only a single guanylate for a locked nucleic acid (LNA) guanylate at the template-switching oligonucleotides (TSOs) 3′-end (rGrG+G) led to a two-fold increase in cDNA library yield, which seems to be a consequence of increased thermal stability of LNA:DNA base pairs. Additionally, the presence of betaine, a methyl group donor, in combination with high (9–12 mM) MgCl_2_ concentrations significantly increased cDNA yield by 2 to 4-fold. What's more, the sensitivity and accuracy was increased by the use of KAPA HiFi Hot Start (KAPA) DNA polymerase, which efficiently amplified the first-strand cDNA directly after reverse transcription with no reduction in cDNA yield, but increased average cDNA length by 450-nt (22, 27). Therefore, compared to Smart-seq, optimized Smart-seq2 with improved reverse transcription, template switching, and pre-amplification increases cDNA library yield and full-length coverage, increases sensitivity and quantitative accuracy, and decreases technical bias.

### T7-Based IVT Amplification and Sequencing

The use of the T7 RNA polymerase to perform linear isothermal amplification of cDNA by IVT, requiring three rounds of amplification, was first reported by Van Gelder in 1990 (28). This strategy has been extensively applied to scRNA-seq in recent years, and helped promote the birth of the era of single-cell analysis (29–31). T7-based IVT amplification was developed for the synthesis of the second-chain cDNA sequences with no need for an anchor sequence. In this method, when the first-chain cDNA is synthesized, the RNA polymerase promoter sequence is led into and cDNA amplification is initiated by an IVT reaction. Currently, there are several scRNA-seq strategies based on T7 RNA polymerase, including cell expression by linear amplification and sequencing (CEL-seq) (29), CEL-seq2 (32), Quartz-seq (33), massively parallel single-cell RNA sequencing (MARS-seq) (30), and indexing droplets RNA sequencing (inDrops-seq) (34, 35). In what follows, we will review them in detail.

#### CEL-seq

CEL-seq is the first method to use IVT that allowed the 3′-end of mRNAs to be detected. Considering that the application of high throughput sequencing was limited by the small initial amounts of RNA, CEL-seq was developed to overcome the limitation by barcoding and pooling samples before linearly amplifying mRNA with the use of one round of IVT (29). This technique is initiated by a single cell reverse transcription reaction using a CEL-seq primer, which is designed with an anchored poly(dT) stretch, a characteristic barcode, an Illumina 5′ sequencing adaptor, and a T7 promoter (29). RNAs from individual cells are uniquely barcoded with a CEL-seq primer for reverse transcription. After the second-strand synthesis, multiple samples are pooled for IVT reactions. Then, the amplified RNAs are fragmented and the reactions are terminated by placing on ice with the addition of EDTA, followed by RNA cleanup before entry into a modified version of the Illumina directional RNA protocol. After 12 cycles of PCR amplification, the fragments with both 5′- and 3′-Illumina adaptors are selected to construct the cDNA libraries, which are sequenced on an Illumina HiSeq2000 via paired-end reads strategy, where the first read recovers the barcode, while the second read identifies the mRNA transcript (24, 29). Compared to Smart-based scRNA-seq, CEL-seq gives rise to more reproducible, linear, and more sensitive outcomes than a PCR-based amplification; these advantages rely on the linear pattern of amplification. What's more, unlike PCR amplification, CEL-seq does not exponentially deplete sequences which are unfavorable to the PCR process (24, 29, 36).

#### CEL-seq2

Based on the technique of CEL-seq, Hashimshony et al. developed a modified version, called CEL-seq2, with higher sensitivity, lower cost, and less hands-on time (32). In this protocol, cell lysis, reverse transcription, the second-strand synthesis, and IVT were performed on-chip. Early barcoding enabled highly-multiplexed analysis, and 3′-end tagging was used, which enables accurate estimation of expression levels without having to account for gene length and with fewer sequencing reads required (32, 37). For CEL-seq2 primers, a 6-base unique molecular identifier (UMI) was integrated into the CEL-seq primer which is located between the barcode and the Illumina 5′ sequencing adaptor. The length of the barcode was reduced from eight to six nucleotides, and the T7 promoter and the Illumina 5′ sequencing adaptor were shortened. The procedures for CEL-seq2 are nearly identical to CEL-seq, except that the sequencing is performed on the Fluidigm C1 system.

The advantages of CEL-seq2 are derived from the use of optimized primers, reagents, RNA cleanup and purification, and library preparation steps. These modifications greatly improved the quality of the data and make CEL-seq2 more time- and cost-efficient. What's more, the Fluidigm C1 system-enabled cell-barcoding allows for a single library construction, instead of setting up multiple library preparations individually (32).

#### Quartz-seq

Emerging Quartz-seq is another scRNA-seq technique that allows highly quantitative gene expression analysis. Quartz-seq is based on polyA tailing, PCR amplification, and IVT, and follows a simpler procedure resulting in higher reproducibility and greater sensitivity surveying the mRNA content of single cells than previously developed methods. Moreover, comprehensive and quantitative detection of gene expression heterogeneity can be achieved by this approach. Quartz-seq was first reported by Sasagawa et al. in 2013 (33). First of all, polyadenylated RNA is reverse transcribed to generate the first strand cDNA using reverse transcription primers that contain oligo(dT), the T7 promoter and PCR target region sequences. After the first-strand cDNA synthesis, the majority of the reverse transcription primer is digested by exonuclease I. Then, a polyA tail is added to the 3′-ends of the first-strand cDNA and any surviving RT primer, and the synthesis of the second strand is initiated with the tagging primer. The resulting cDNA and the byproducts from the surviving primers contain the whole-transcript amplification adaptor sequences, which include the reverse transcription primer sequence and the tagging primer sequence. Subsequently, these DNAs are used for suppressing PCR with the suppression PCR primer. After fragmenting the amplified cDNA using the Covaris shearing system and enriching the short cDNA fragments, the high-quality cDNA libraries are obtained. Finally, the cDNA sequencing libraries are analyzed by an Illumina sequencer (33). In this protocol, only six reaction steps are required and all steps are completed in a single PCR tube without any purification, which dramatically simplifies the Quartz-seq method.

#### MARS-seq

To construct single cell libraries from polyadenylated RNA, a new method, termed MARS-seq was developed by Jaitin et al. in 2014 (30). Briefly, single cells are isolated by FACS into 384-well plates containing hypotonic cell lysis buffer and reverse transcription primers. The reverse transcription primer consists of anchored poly(dT), UMI, cell barcode, partial Illumina paired-end primer sequence, and a T7 RNA polymerase promoter. The cell barcode is used for subsequent de-multiplexing, and UMI for correction of amplification biases (30, 37). After the first-strand cDNA synthesis, exonuclease I is added and samples are pooled for the second-strand synthesis. Subsequently, samples are linearly amplified by IVT and chemically fragmented into a median size of ~200 nucleotides. Then, a partial Illumina Read1 sequencing adapter including a pool barcode is single strand ligated to the fragmented RNA using a T4 RNA ligase I. The ligated product is reverse transcribed and the cDNA is purified. Next, the sequencing library is completed and amplified through a nested PCR reaction, with the forward primer containing the Illumina P5-Read1 sequences and the reverse primer containing the P7-Read2 sequences. Finally, MARS-Seq libraries are paired-end sequenced by an Illumina HiSeq 2500 (30). This protocol is based on FACS-sorting of single cells into 384-well plates and subsequent automated processing on a Bravo robot station, and is generally performed on pooled and labeled material, leading to significant increases in throughput and reproducibility.

#### InDrops-seq

Droplet microfluidics have been used to develop a technique for indexing thousands of individual cells for RNA sequencing, termed indexing droplets (inDrops) RNA sequencing; this technique has the capability to index >15,000 cells per hour, boasts low technical noise, and is adaptable to other sequencing-based assays (34, 35). Briefly, isolated individual cells are encapsulated into nanoliter droplets containing lysis buffer, reverse-transcription mix, and reverse transcription oligonucleotide primers consisting of poly(dT), UMIs, cell barcodes, sequencing adaptors, T7 RNA polymerase promoters, and photocleavable spacers linked to hydrogel microspheres (35). After encapsulation primers are released by photocleavage (i.e., 8-min exposure to >350-nm UV radiation), cDNA synthesis is initiated, and the synthesized cDNAs remaining trapped in each droplet are tagged with a barcode during reverse transcription. After synthesis of the second-strand cDNA, linear amplification is carried out by IVT. Subsequently, encapsulated droplets are broken and the amplified antisense RNA is fragmented using zinc-ion-mediated cleavage. Cleaved RNA is converted into a cDNA library for next-generation sequencing by a second reverse transcription reaction and a few cycles of PCR. Finally, the cDNA libraries are sequenced on Illumina sequencing platforms (34, 35). This general strategy used follows that developed by Hashimshony et al. (29) and Jaitin et al. (30), but several steps have been added or revised. For example, in this methodology, the 3′-5′ exonuclease ExoI and dsDNA-specific restriction endonuclease HinfI are used to digest unused ssDNA primers and cleaves the T7 promoter sequence in primer dimers and hairpins, respectively (35).

Recently, a novel method was developed that combines exponential amplification of a limited number of PCR cycles with linear amplification of T7-based IVT for RNA amplification (38). The novelty of this technique lies in that it allows for high-fidelity gene expression profiling of individual cells and for significant reduction of 3'-bias; this enables detection of all regions of RNA transcripts. To achieve this objective, some protocol modifications have been made. These modifications include: (1) the introduction of “extending primers” that contain a Kozak sequence at the 3′-ends during PCR which allows capture of 5′-ends of a gene's coding sequence; (2) the combination of modified polyT and modified random primers to make the PCR step more efficient and to improve the preservation of relative gene abundance, which secures full-length RNA coverage, and eliminating carryover of reverse transcriptase that greatly diminishes reverse transcriptase inhibitory effect on the following PCR steps; (3) using a SmartSpec 3000 spectrophotometer (Bio-Rad), the RNA yield was evaluated, and the yield of amplified RNA came up to 200–250 μg from a single cell, which is sufficient to apply to any RNA sequencing technique (38). In a word, this new technique has great potential for future applications.

### Phi29 DNA Polymerase-Based Amplification

Derived from the *Bacillus subtilis* phage Phi29, the Phi29 DNA replicative polymerase has strong strand displacement activity (39). Phi29 DNA polymerase based RNA amplification has been successfully applied to construct cDNA libraries from single prokaryotic (40) and eukaryotic cells (23). Reverse transcribed single strand cDNA tends to self-ligate and to amplify like a rolling circle in the presence of Phi29 DNA polymerase, so Phi29 DNA polymerase-based amplification is also referred to as rolling circle amplification.

#### PMA

Phi29 DNA polymerase-based mRNA transcriptome amplification (PMA), an approach for single and low quantities cell cDNA amplification, is the first strategy using Phi29 DNA polymerase to construct cDNA libraries for the sequencing of whole mRNA transcriptomes. The PMA method allows for full-length transcript coverage to be obtained. Methods for multiple displacement amplification of circularized cDNA have been discussed in detail by Liu and her colleagues (24).

#### TTA

Another single-cell RNA amplification method based on Phi29 DNA polymerase is total transcript amplification (TTA) (40). This transcript amplification method involves multiple rounds of PCR and/or linear amplification of cDNA and is challenged by low amounts of RNA and lack of polyA-tails for easy tagging and mRNA specific amplification (5, 33, 40). However, the TTA strategy yields reproducible data, low fold-change bias, and a high number of genes efficiently amplified (40).

### MALBAC-RNA

In addition to the above methods, multiple annealing and looping based amplification cycles (MALBAC) is also applied to single-cell transcriptome amplification (termed MALBAC-RNA). MALBAC-RNA was developed and first used in whole genome amplification by Xie et al. (41, 42). This strategy boasts high detection efficiency, accuracy, and reproducibility. After cell lysis and reverse transcription, primers with 7 random nucleotides are annealed to the synthesized first-strand cDNA and are then extended by DNA polymerase with strand displacement activity. Amplicons are then melted off the original template after extension, and are looped to protect themselves from being further amplified. This MALBAC-RNA step includes a total of 10 cycles of quasilinear amplification (MALBAC-RNA pre-amplification), followed by another 19 cycles of PCR. Finally, these MALBAC amplified DNA products are directly used for sequencing library preparation and are then sequenced on an Illumina HiSeq 2000 instrument (42).

Recently, several other strategies have been exploited for single-cell transcriptome amplification and sequencing, including multiple annealing and dC-tailing based quantitative single-cell RNA sequencing (MATQ-seq) (43), CRISPR droplet sequencing (CROP-seq) (44), and geographical position sequencing (Geo-seq) (45).

### MATQ-seq

MATQ-seq is a highly sensitive and quantitative method for single-cell sequencing of total RNA, including noncoding and nonpolyadenylated RNA, and was inspired by the primers used in MALBAC (43). In MATQ-seq, reverse transcription is performed with a primer that consisting of the MALBAC primer, three consecutive G or T, and 20 consecutive T (dT_20_). This primer design differs from MALBAC, in that it mainly contains G, A, and T bases. After 9 cycles of reaction, primers and RNAs are digested by T4 DNA polymerase and RNase H and RNase I, respectively. Then, polyC tailing is performed on the cDNA, and the second-strand synthesis initiates with G-enriched MALBAC primers, containing a random hexamer UMI sequence (referred to as amplicon indexes); the use of these primers decreases the 3′- or 5′-end bias to a great extent. After amplification, Illumina Truseq adaptors are ligated to the samples, purified cDNA libraries are pooled as instructed by Illumina protocol, and libraries are sequenced on the Illumina Nextseq 500 platform (43).

### CROP-seq

Combining pooled CRISPR screening with scRNA-seq, a new method called CROP-seq was developed. CROP-seq involves directly linking guide-RNA (gRNA) expression to transcriptome responses in thousands of individual cells (44). In this strategy, a gRNA vector (LentiGuide-Puro) is introduced, because CRISPR gRNA lacks a polyA tail and is not detectable with current scRNA-seq assays, which makes CROP-seq compatible with previously developed scRNA-seq methods. A detailed protocol was reported in the *Journal of Nature Methods* (44). Briefly, single cells are co-encapsulated with barcoded beads and resuspended in Drop-seq lysis buffer. Reverse transcription and exonuclease I treatment are then performed, and an additional ten enrichment cycles using the Illumina Nextera XT i7 primers, along with the Drop-seq New-P5 SMART-PCR hybrid oligonucleotides, are performed to enrich the cDNA library. Finally, cDNA libraries are sequenced with paired-end SBS chemistry on using an Illumina HiSeq 3000/4000 instrument. Using sophisticated molecular technologies, Datlinger's team constructed a viral vector that can help researchers see the CRISPR gRNA in single-cell sequencing (44). With this technique, thousands of genome editing events in single cell were analyzed in a high throughput manner; this will facilitate high-throughput functional dissection of complex regulatory mechanisms and heterogeneous cell populations.

### Geo-seq

*In situ* position information of cells is lost by current single-cell sequencing strategies. However, the native spatial information is critical to some research fields, such as developmental biology and tumor biology. Driven by this necessity, a novel single-cell transcriptome sequencing technique called Geo-seq was exploited. Geo-seq combines LCM and scRNA-seq technology (45). In this approach, LCM-sorted single cells are lysed for first-strand cDNA synthesis, and a modified Smart-seq2 protocol is performed to amplify the cDNA. Of the many scRNA-seq methods, Smart-seq2 was chosen for the ability of achieving full-length cDNA synthesis with a low amplification bias in a short time period (27, 46). Geo-seq is also compatible with Brady/Tang's method (5, 19). cDNAs that pass quality control are then used to construct the library for next-generation sequencing (45). As an alternative method, Tomo-seq has been devised for spatial gene profiling (47). Geo-seq is a high-efficiency, high-resolution strategy for spatial transcriptome analysis, which can be used in 3D reconstruction of the transcription profile, and can also be used to study the transcriptome information of a small amount of tissues or cells with special structures. The Geo-seq technique shows wide application potentials to address biological and pathological questions of cellular properties such as prospective cell fates, biological functions and gene regulatory networks (45).

### Split-Pool Ligation-Based Transcriptome Sequencing

Amazingly, a latest article published in *Science* reported a low-cost scRNA-seq method, called “split-pool ligation-based transcriptome sequencing” (SPLiT-seq) (48). In SPLiT-seq, four rounds of combinatorial barcoding are applied to an individual transcriptome. In the first round, formaldehyde fixed cells are added into a well-plate, and in-cell reverse transcription is accomplished using well-specific barcoded primers. Next, all cells are collected and redistributed into a new well-plate, where an in-cell ligation reaction appends a second well-specific barcode to the transcribed cDNA. The third round of barcoding applies barcodes containing unique molecular identifiers (UMIs). After that, the cells are pooled, divided into sublibraries, and sequencing barcodes are introduced by PCR; meanwhile, the fourth barcodes are led into. Finally, after paired-end sequencing on a MiSeq or NextSeq system (Illumina), each individual transcriptome is assembled by combinatorial reads that combine the same four barcodes (48).

**Figure 1:**
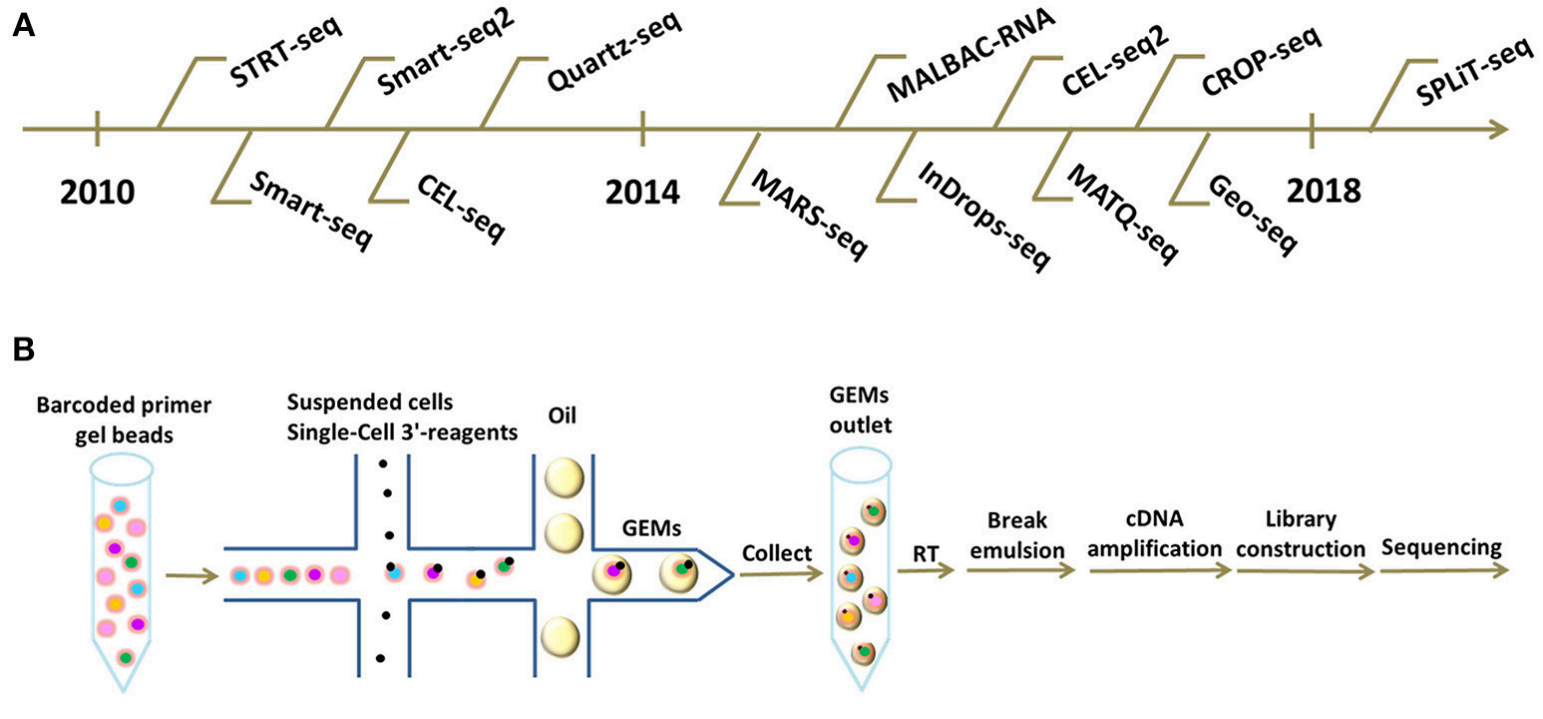
Different scRNA-seq technologies and frontier cDNA library construction platforms. **(A)** Chronological chart of existing scRNA-seq technologies. **(B)** Workflow of scRNA-seq based on 10X Genomics platform. Briefly, suspended cells and Single-Cell 3'-reagents are added into the microfluidics chip. Subsequently, together with barcoded primer gel beads and oil-surfactant solution, GEMs are formed in a “double cross” way. Single GEMs are collected in the GEMs outlet. Next, reverse transcription is initiated inside each GEM. Afterwards, GEMs are broken and barcoded cDNAs are pooled for amplification and library construction.

SPLiT-seq is compatible with fixed cells or nuclei, and requires no partitioning of single cells into individual compartments, but completely relies on the cells themselves as compartments. This novel technique labels the cellular origin of RNA through combinatorial DNA barcodes only with pipetting steps and requires no complex apparatus, which greatly simplify the workflow, decreases the cost, and widely facilitates the scalable profiling of single cells (48). As this method emerges as a viable sequencing technique, we believe that the cost of scRNA-seq will be greatly reduced and the application of scRNA-seq will become more efficient and widespread. A chronological chart of all these existing scRNA-seq technologies listed above is shown in Figure 1A.

### Frontier cDNA Library Preparation Platforms in Single-cell RNA-seq

For years, Illumina systems have been regarded as the leaders in the field of cDNA library construction in scRNA-seq. However, at the Advances in Genome Biology and Technology (AGBT) meeting in 2015, the GemCode platform based on 10X Genomics was officially launched, which immediately raised great interest in the research community. A year later, Chromium™ single cell 3′-solution, based on 10X Genomics platform as an updated version of GemCode platform, was launched. The Chromium™ methodology greatly accelerates the applications of scRNA-seq and assists digital single-cell transcriptome sequencing analysis. Within 3 years, scRNA-seq based on the 10X Genomics platforms has exploded in popularity (49–60).

Single-cell transcriptome sequencing, using the 10X Genomics platforms, can obtain expression information from 1,000 to 10,000 cells simultaneously, and can analyze the grouping of cells and detect gene expression differences between cell populations. This method is currently regarded as the “golden method” to evaluate tumor heterogeneity, immune cell populations, and embryonic development.

The workflow on 10X Genomics platform is summarized below (Figure 1B). Initially, suspended cells are combined with GemCode Single-Cell 3′ reagents in one channel of an 8-channel microfluidics chip. Subsequently, millions of barcoded primer gel beads and oil-surfactant solution from other channels are introduced to form GEMs (Gel Bead-In-Emulsions) in a “Double Cross” way, GEMs containing single cells are then collected in the GEM outlet. Next, reverse transcriptions are initiated inside each GEM with primers containing Illumina adaptors, 10X barcodes, UMIs, and oligo(dT) sequences. Afterwards, the GEMs are broken and barcoded cDNAs are pooled for amplification and library construction (59). GEM formation is the core of this technology and is vital to the success of scRNA-seq.

10X Genomics platforms are high throughput technologies that agglomerate single-cell isolation, cDNA amplification and library construction into one process, which greatly reduces sample manipulation and saves cost and time. Moreover, the 10X Genomics platform boasts up to 65% single cell capture rate, and cell suspension preparation, single cell capturing, cDNA amplification, and library construction can be achieved within a single day. Taking these advantages into consideration, we believe that 10X Genomics platforms will have wide application prospects in the field of scRNA-seq.

## Single-Cell RNA Sequencing Applied in Normal Hematopoiesis

The hematopoietic system is well established as a paradigm for studying cellular hierarchies. Traditional approaches to study hematopoiesis involve purification of cell populations based on a series of surface markers and functional assays; these assessments obscure underlying heterogeneity contained within any phenotypically defined cell population (61). In recent years, advances in scRNA-seq have opened new avenues to characterize heterogeneity in a large variety of cellular systems, and will lead to the discovery of novel subpopulations (62–65).

### Single-Cell RNA Sequencing in HSPCs

Tang et al. demonstrated that by applying both Smart-seq2 and InDrops scRNA-seq approaches to define cell heterogeneity, they were able to identify a group of classically defined and erythroid-primed hematopoietic stem and progenitor cells (HSPCs) (62). What's more, Paul et al. identified 18 unrecognized subpopulations with distinct lineages priming toward specific lineage fates, as compared to the traditional definition of myeloid progenitors by analyzing HSPCs by scRNA-seq (63). They discovered that Cebpa and Cebpe are two cell fate decision transcription factors found in myeloid progenitors. High levels of Cebpa expression direct myeloid progenitors to neutrophil, monocyte, and basophil lineages, whereas high levels of Cebpe expression seem to be found primarily in neutrophil and eosinophil subpopulations (63). Thus, scRNA-seq was recommended as an ideal tool for demarcating developmentally plastic and highly dynamic cell populations in hematopoiesis. Additionally, applying scRNA-seq to both standard HSC and their progeny multipotent progenitor 1 (MPP 1), three main clusters with distinct features were separated, including active, quiescent, and a novel, undefined fraction of cells (64). Furthermore, Kowalczyk et al. found extensive transcriptome variability among HSCs assessed by scRNA-seq (66). Additionally, scRNA-seq is able to determine lineage heterogeneity among stem cell compartments. Tsang et al. applied scRNA-seq to 180 highly purified *Bcl11a*^−/−^ and *Bcl11a*^+/+^ HSCs, and found that *Bcl11a*^−/−^ HSCs consist of two distinct myeloerythroid-restricted subpopulations (65). Moreover, scRNA-seq was used to distinguish aneuploid cells from diploid cells within the hematopoietic stem and progenitor cells. Zhao et al. performed scRNA-seq on bone marrow-derived CD34^+^ cells from patients with bone marrow failure and cytogenetic abnormalities, distinguishing aneuploidy hematopoietic stem and progenitor cells from diploid cells (67).

In addition, scRNA-seq was successfully applied to tracing the formation of HSCs (68, 69). In addition to haemogenic endothelial cells, HSCs are derived from pre-HSCs, which consist of at least two HSC-competent intermediates around embryonic day 11 (E11), namely, CD45^+^ pre-HSCs and CD45^−^ pre-HSCs. Applying scRNA-seq to CD45^+^ and CD45^−^ pre-HSCs in E11 mouse aorta-gonad-mesonephros, Zhou *et al*. revealed that there existed a remarkable heterogeneity in relevant populations during the stepwise development of HSC from the pre-HSC stage, and showed a continuous development from endothelial cells to HSCs through CD45^+^ and CD45^−^ pre-HSCs (68). Autophagy plays an essential role in self-renewal and differentiation in embryonic hematopoiesis. Recently, Hu et al. applied scRNA-seq to five cell populations related to HSC formation and reported that the transcription activity of autophagy-related genes was substantially increased when endothelial cells committed to pre-HSCs, suggesting that autophagy plays a critical role in HSC formation (69).

scRNA-seq has also been widely applied to hematopoietic cell differentiation (70–74). HSCs sit at the very top of a differentiation hierarchy, which produces the full spectrum of mature blood cells via intermediate progenitors (70). Using scRNA-seq, Nestorowa et al. profiled 1,656 single HSPCs to reconstruct differentiation trajectories, and revealed the dynamic expression changes associated with early lymphoid, erythroid, and granulocyte-macrophage differentiation (70). This enabled the reconstruction of an atlas showing the differentiation trajectories of HSPCs. Additionally, with phenotypic and functional assays, at least four subpopulations were revealed within the B220^+^CD117^int^CD19^−^NK1.1^−^ uncommitted hematopoietic progenitor, of which the Ly6D^+^SiglecH^−^CD11c^−^ subtype exhibited strong B-cell potential, whereas the Ly6D^−^SiglecH^−^CD11c^−^ subtype showed mixed lympho-myeloid potential (71). Applying scRNA-seq to the Ly6D^+^SiglecH^−^CD11c^−^ subtype, Alberti-Servera et al. identified a new subset, which was thought to be the direct precursor of the first B-cell committed stage (71). Additionally, combining index FACS sorting with computational reconstruction of thrombocyte developmental chronology from scRNA-seq data, Macaulay et al. revealed the continuous nature of thrombocyte lineage commitment from stem cells to mature cells (72). Similarly, Velten et al. applied scRNA-seq to human CD34^+^ HSPCs, and showed that human HSC lineage commitment is a continuous process (73). In another study, 2000 HSCs from young and old *Vwf-EGFP* mice were isolated, respectively, and 61-young and 74-old HSCs were captured and prepared for scRNA-seq. Researchers identified increased molecular platelet priming and functional platelet bias as the predominant age-dependent change to HSCs, including a significant increase in a previously unrecognized class of HSCs, a Vwf^+^ platelet-biased HSC subpopulation, that exclusively produce platelets, which demonstrated that there exists molecular and functional platelet bias in aged HSCs (74). Additionally, they found that FOG-1 is an intrinsic regulator of Vwf^+^ platelet-biased HSC subset for the first time. Data shows that FOG-1 is critical to the maintenance of the Vwf^+^ platelet-biased HSC subset (74).

scRNA-seq has also been applied to evaluate the regulation of signaling pathways in HSCs (65, 74, 75). For example, Cabezas-Wallscheid et al. applied scRNA-seq to determine vitamin A in the maintenance and function of HSCs, they uncovered a role for vitamin-A retinoic acid signaling in the regulation of HSC dormancy, and they reported that the transition from dormancy toward cell-cycle entry is a continuous developmental path associated with upregulation of biosynthetic processes rather than a stepwise progression (75). Furthermore, applying scRNA-seq to *Bcl11a*^−/−^ and *Bcl11a*^+/+^ HSCs, Tsang et al. discovered that transcription rates periodically increase from G1 to S/G2/M phase, suggesting that *Bcl11a* deficiency is associated with increased proliferation in the HSC compartment (74). In these cases, it was demonstrated that scRNA-seq is a powerful approach to identify novel intrinsic or extrinsic regulators of HSC function.

scRNA-seq has been applied to identify distinct myeloid progenitor differentiation pathways, and has the potential to simultaneously resolve cellular heterogeneity and to identify the molecular determinants that are selectively and uniformly expressed in novel subgroups (76). Lymphoid-primed multi-potent progenitors (LMPPs) and common myeloid progenitors (CMPs) are regarded as the first branch point for separating lineage commitment pathways from HSCs. However, it has been proposed that both differentiation pathways generate myeloid innate immune cells through the same myeloid restricted pre-granulocyte macrophage progenitor (pre-GMP). Applying scRNA-seq to pre-GMPs, Drissen et al. identified two distinct myeloid differentiation pathways, namely the *Gata1*-expressing pathway, which generates mast cells, eosinophils, megakaryocytes, and erythroid cells, and the *Gata1*-negative pathway, which generates monocytes, neutrophils, and lymphocytes (76).

scRNA-seq can provide a detailed molecular characterization of single cells, and is highly complementary to traditional differentiation or FACS-based phenotyping approaches (77). To individually profile 20,000 hematopoietic progenitor cells, without prior enrichment or depletion of individual lineages based on surface markers from human cord blood, a massively parallel scRNA-seq approach, Drop-seq, was applied; this approach identified intermediate stages that simultaneously co-express “primed” programs for multiple downstream lineages and revealed striking heterogeneity in the early molecular transitions between myeloid subsets during human cord blood hematopoiesis (78).

### Single-Cell RNA Sequencing in Lymphoid Cells

Lymphoid cells are derived from HSCs, and are mainly responsible for body immunity. scRNA-seq has rarely been applied to studying lymphoid cells in hematopoiesis. In one example, Yu et al. applied scRNA-seq to bone marrow innate lymphoid cells (ILCs); they identified distinct ILC progenitor subsets, including a novel programmed death 1 high expression (PD-1^hi^) ILC progenitor subset, and delineated distinct ILC developmental states and pathways (79). In another example, Patil et al. applied scRNA-seq to more than 9000 CD4^+^ T cells from the peripheral blood of healthy donors to unravel heterogeneity among CD4^+^ cytotoxic T lymphocytes (CD4-CTLs), and to evaluate the transcriptional profiles and clonality within the population; they identified that CD4-CTL is derived from a subset of effector memory T cells expressing CD45RA(T_EMRA_) (80). Identification of the CD4-CTL precursor population may enable further research of how CD4-CTLs arise, and could elucidate the mechanisms that may be applied to generate durable and effective CD4-CTL immunity (80).

### Single-Cell RNA Sequencing in Myeloid Cells

#### Dendritic Cells

Dendritic cells (DCs) are immune cells that are differentiated from granulocyte monocyte progenitors (GMPs). The main functions for DCs are antigen sensing and presentation. Little is known about the transcriptional heterogeneity within cells in responses to antigens. scRNA-seq was used to investigate heterogeneity in the response of bone-marrow derived de DCs to lipopolysaccharide(LPS), and revealed an extensive, bimodal variation in mRNA abundance and splicing patterns, which demonstrated previously unobserved levels of heterogeneity between DCs (81). What's more, Shalek et al. used scRNA-seq to show that bone-marrow derived DCs, stimulated under different conditions *in vitro*, demonstrate variable responses that are mediated by interferon paracrine signaling (82). Additionally, using scRNA-seq, Villani et al. identified six human DC clusters, including two clusters mapping closely to the well-established DC subsets and four new clusters. These newly identified DC subsets included one that shares properties with plasmacytoid dendritic cells (pDCs) but potently activates T cells, redefining the roles for pDCs (83).

Generally speaking, the expression of transcription factors and abundance of surface proteins determine cell fate specification during differentiation. Applying scRNA-seq to mouse bone marrow conventional dendritic cells (cDCs), Papalexi et al. identified that Siglec-H and Ly6C abundance determines whether cDCs will become cDC type 1 (cDC1, Siglec-H^−^Ly6C^−^) or cDC type 2 (cDC2, Siglec-H^−^Ly6C^+^) (84).

#### Monocyte

scRNA-seq has also been applied to identifying heterogeneity within monocyte populations. Villani et al. sequenced 372 single blood monocytes by scRNA-seq, and identified four monocyte subsets, including two known and two new clusters (83). In addition, 1,700 suspended human monocytes were sequenced by 10X Genomics Chromium, revealing differences in monocyte differentiation fate; specifically, IRF4 and MAFB were found to be essential for differentiation of monocyte-derived dendritic cells (mo-DCs) and differentiation of monocyte-derived macrophages (mo-Macs), respectively. Moreover, aryl hydrocarbon receptor activation promotes differentiation of mo-DCs and inhibits differentiation of mo-Macs through BLIMP-1(52).

**Figure 2:**
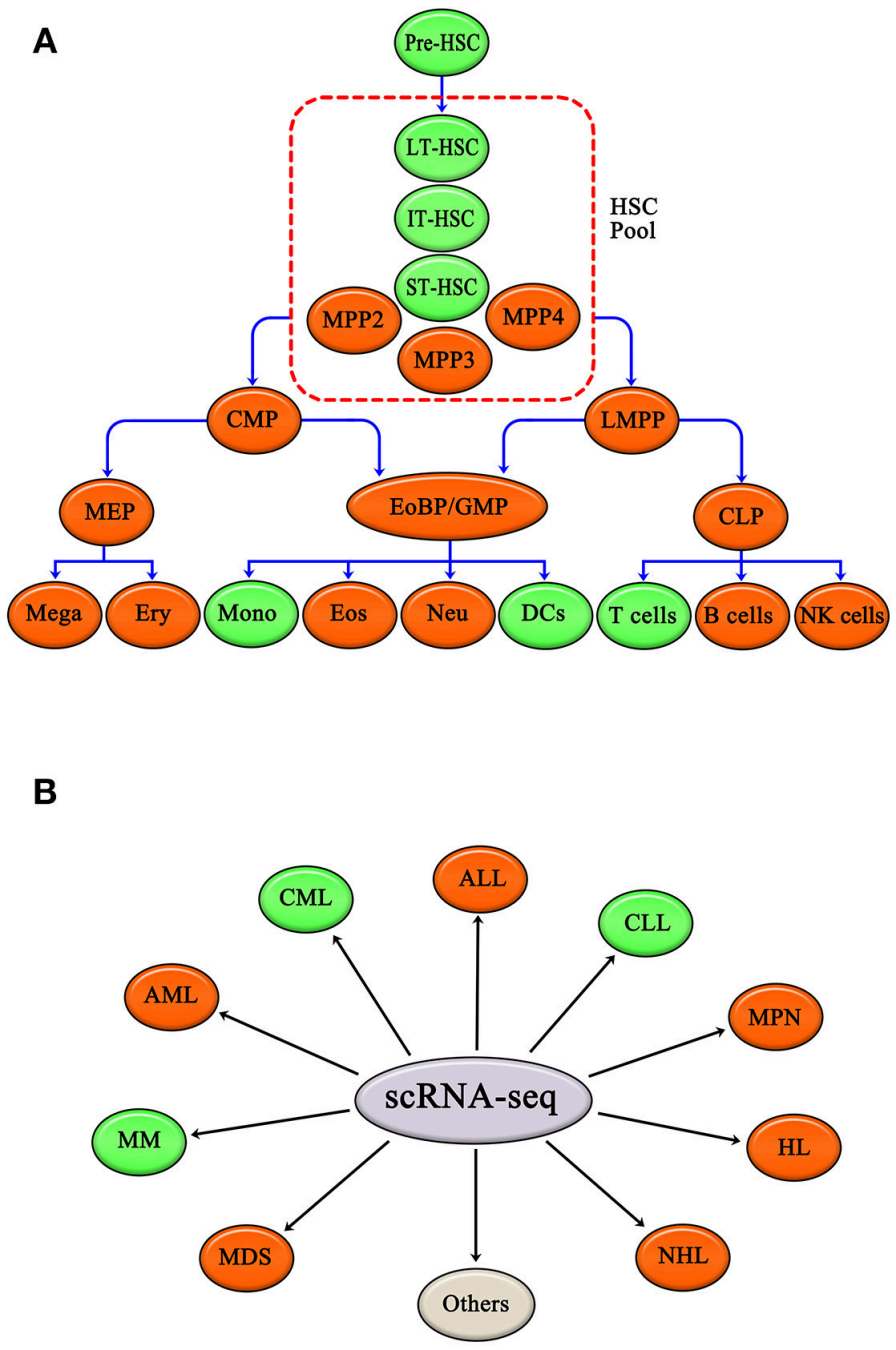
Normal hematopoietic cells and malignant hematopoiesis have been studied by single cell RNA-seq. **(A)** Single cell RNA-seq applied to hematopoietic cells, such as HSC, DCs, Mono, and T cells. Other hematopoietic cells, like CMP, GMP, MEP, LMPP, CLP, Mega, Ery, Eos, Neu, B cells, and NK cells, are also expected to be researched. **(B)** Single cell RNA-seq has been applied in CML, CLL, and MM. Single cell RNA-seq is expected to be applied to other hematopoietic malignancies as well, including AML, ALL, MPN, HL, NHL, and MDS. Green ovals show normal hematopoietic cells and malignant hematopoiesis that have already been studied by single cell RNA-seq, and red ovals show hematopoietic cells and malignant hematopoiesis that have yet to be evaluated by scRNA-seq. Gray oval represents other hematopoietic malignancies apart from those listed above.

To sum up, scRNA-seq has been applied to study various hematopoietic cells, including HSCs, DCs, monocytes, and T cells. Other hematopoietic cells, such as common myeloid progenitors (CMP), granulocyte-monocyte progenitors (GMP), megakaryocyte-erythroid progenitors (MEP), lymphoid-primed multipotential progenitors (LMPP), common lymphoid progenitors (CLP), megakaryocytes, erythrocytes, eosinophils, neutrophils, B cells and natural killer cells (NK cells), are also expected to be studies using scRNA-seq (Figure 2A).

## Single-Cell RNA Sequencing Applied in Malignant Hematopoiesis

Apart from its broad use in studying physiological hematopoiesis, scRNA-seq has also been extensively applied to malignant hematopoiesis. Leukemia, lymphoma, multiple myeloma (MM), myeloproliferative neoplasm (MPN), and myelodysplastic syndrome (MDS) are the common hematopoietic malignancies which seriously threaten health and even the survival of patients. Currently, scRNA-seq has been used to study leukemia (85, 86) and multiple myeloma (87) in order to better understand these diseases and find more effective therapeutic strategies.

Combining high sensitivity mutation detection with scRNA-seq, Giustacchini et al. analyzed more than 2000 stem cells (SCs) from patients with chronic myeloid leukemia (CML), revealing previously unrecognized heterogeneity of CML-SCs, including the identification of a subset of CML-SCs with a distinct molecular signature that selectively persisted during prolonged therapy. Furthermore, a blast-crisis-specific SC population was identified, which was also present in a subclone of CML-SCs during the chronic phase (85).

scRNA-seq has also revealed transcriptional heterogeneity in chronic lymphoid leukemia (CLL). Wang et al. performed single-cell whole-transcriptome sequencing of CLL samples, and identified significant transcriptional heterogeneity representing a variety of cellular processes, including cell cycle, immune signaling, antigen presentation, phospholipid binding, and protein folding (86). Furthermore, they combined scRNA-seq with targeted mutation detection and identified *LCP1* and *WNK1* mutations as novel CLL driving events (86).

Moreover, scRNA-seq has been applied in MM to accurately identify chromosomal translocations and to facilitate differentiation of normal plasma cells from malignant cells in an unbiased fashion (87). Unsupervised hierarchical clustering of MM cells between two patients revealed different subtypes of MM cells. Chromosomal translocations, such as the t(11;14) translocation and t(6;14) translocation, are important markers for stratifying the clinical risks of MM (88). Using single circulation tumor cell (CTC) RNA-seq to identify chromosomal translocations, Lohr et al. identified which genes are the most differentially expressed between single MM CTCs and normal B lymphocytes, suggesting that single CTC gene expression profiling may be applied for the diagnosis and classification of MM (87). scRNA-seq has the potential for great clinical utility and can elucidate precision medicine approaches for the treatment of relapsed/refractory MM.

Additionally, scRNA-seq could also be used to identify putative leukemia stem cell populations based on “stemness” signatures, using available data (89), or to predict *de novo* stem cell identities from scRNA-seq data, using recent evolutionary algorithms (90).

scRNA-seq has been applied to several hematopoietic malignancies, including CML, CLL, and MM; and other malignancies, including acute myeloid leukemia (AML), acute lymphoid leukemia (ALL), MPN, Hodgkin's lymphoma (HL), non-Hodgkin's lymphoma (NHL), and MDS, are likely to be evaluated by scRNA-seq in the future (Figure 2B).

## Outlook

Hemetopoiesis is the process of development of normal hematopoietic cells, which is an ordered multi-step process originated from a rare population of multipotent and self-renewing HSCs within the bone marrow. During malignant hematopoiesis, including leukemias, MDS, and MM, et al., the uncontrolled proliferation and blocked differentiation of precursors sustains malignancy growth at the expense of normal blood cell production in bone marrow. Normal and malignant hematopoiesis is perhaps the best-defined hierarchy model and has led the way in the application of new single-cell approaches to unravel cellular heterogeneity.

Before the advent of scRNA-seq, discovering novel cell subsets in normal and malignant hematopoiesis mainly relies on cell surface markers via FACS-sorting, which required prior knowledge or informed guesswork. The advances in high-dimensional, single-cell techniques have since enabled unbiased, alternative workflows to sequence cells with no need for prior knowledge of genes and proteins, and grouping of cells is accomplished based on their transcriptional signatures (84). So far, scRNA-seq has shown great effectiveness in unraveling complex cell populations, reconstructing developmental trajectories, and modeling transcriptional dynamics in but not restricted to normal and malignant hematopoiesis (91).

Cellular heterogeneity is present in nearly every organ and tissue, as well as tumors, which hinders efforts to unravel cellular functions and the roles of different cell populations, and presents a challenge to the development of precision medicine. scRNA-seq is a powerful tool to reveal cell-to-cell variation and cellular heterogeneity, discover new cell types and characterize malignant hematopoiesis and other tumorigenesis (92). Over the years, cellular heterogeneity has been evaluated in many tissues and cells using scRNA-seq (93–103). scRNA-seq has been applied to solve heterogeneity within hematopoietic cells as well (63, 64, 79, 81, 83). Additionally, RNA sequencing combined with single-cell network analysis has identified DDIT3 as a nodal lineage regulator in hematopoiesis (104), indicating that scRNA-seq and network analysis will advance our knowledge of hematopoiesis. Subsequent bioinformatics analysis of scRNA-seq data can be used to identify key genes involved in embryonic development, immune responses, and carcinogenesis. The maturation of scRNA-seq techniques has made it easier to evaluate gene expression profiles at single-cell and single-base resolution (21, 105). More recently, Athanasiadis et al. applied scRNA-seq to uncover transcriptional states and fate decisions in hematopoiesis, to order cells along their differentiation hierarchy, and to computationally reconstruct the blood lineage tree (106). scRNA-seq has been applied to guide the diagnosis and treatment of the patients with malignant hematological diseases in recent years. scRNA-seq of CML-SCs at diagnosis of patients predicts molecular response to tyrosine kinase inhibitor treatment (85). In addition, CLL scRNA-seq reveals transcriptional heterogeneity and uncovers mutated *LCP1* and *WNK1* as novel CLL drivers (86), they are likely to be novel drug targets for the treatment of CLL. Furthermore, scRNA-seq enables classification of MM (87), which is vital to future personalized therapy and precision medicine.

Besides scRNA-seq, single-cell qPCR has also been used to study hematopoiesis. Moore et al. performed transcriptional profiling of single hematopoietic cells by single-cell qPCR, and analyzed normal, aberrant, and malignant hematopoiesis in zebrafish (107). Compared to scRNA-seq, single-cell qPCR has no need for special and expensive reagents and instruments. However, it relies on specific primer design and is limited to detect known target genes, which introduces a source of subjectivity. Additionally, limited by the amount of RNA, only a dozen of highly expressed genes can be detected by single-cell qPCR. Worse still, it is prone to false positive findings and only suitable for a small number of cells. Compared to scRNA-seq, traditional RNA-sequencing ignores the cell-to-cell variation and cannot truly reflect natural heterogeneity, but it largely promotes our understanding of hematopoiesis before single-cell technique emerged.

As reviewed above, traditional views of the hematopoietic hierarchy have been challenged, modified, enriched, and redefined by scRNA-seq. This new information is leading us to major conceptual shifts and more precise and unambiguous understanding of hematopoietic system, including, but not limited to, hematopoietic cells fate decisions. In addition, gene expression network regulation of hematopoiesis has been elucidated using scRNA-seq (64, 74, 75). Deeper knowledge of regulation of hematopoiesis is helpful to better understand hematopoietic malignancy, and will facilitate new drug research and development. Additionally, scRNA-seq can be applied to other hematopoietic progenitors and mature blood cells, including CMPs, GMPs, MEPs, LMPP, CLPs, megakaryocytes(Mega), erythrocytes(Ery), Eosinophils(Eos), Neutrophils(Neu), B cells, and NK cells (Figure 2A). In addition, scRNA-seq is expected to be applied to other malignant hematological system diseases as well, including AML, ALL, HL, NHL, MPN, and MDS (Figure 2B).

Nevertheless, it still remains a challenge to hone and refine scRNA-seq techniques. A critical step in scRNA-seq is whole transcriptome amplification (WTA), which comprises reverse transcription of mRNA into cDNA, followed by cDNA amplification. Currently, there are three main cDNA amplification approaches: PCR-based amplification, T7-based IVT amplification, and Phi29 DNA polymerase-based amplification. The advantages and disadvantages of these techniques are summarized in Table 2. Briefly, PCR-based amplification of cDNA, being exponential in nature, is prone to introduce variability and discrepancies, especially among low-abundance transcripts (21). T7-based IVT cDNA amplification is a linear amplification method, which will theoretically reduce amplification bias compared to PCR-based methods (29). However, IVT amplification shows a very high 3′-end bias and fails to detect the full spectrum of transcripts, whereas PCR-based protocols are capable of amplifying full-length cDNA. Phi29-based amplification strategies are suitable for low cDNA amplification, and can amplify full-length transcripts, but genomic DNA (gDNA) must be removed before amplification (24). The most appropriate scRNA-seq method should be selected according to the specific experimental requirements.

**Table 2:** The advantages and disadvantages of three main single-cell cDNA amplification methods.

**Amplification methods**	**Advantages**	**Disadvantages**	**References**
PCR-based amplification	Full-length transcript	Introduce variability Introduce discrepancy	(21)
T7-based IVT amplification	Low amplification bias	High 3'-end bias Unable to detect full transcript	(29)
Phi29-based amplification	Low quantity of cDNA Full-length transcript	Remove genomic DNA firstly	(24)

Researchers are exploring how best to analyze the vast quantities of sequencing data that these methods produce, and which algorithm is the most useful. Challenges remain regarding comparing gene expression sequencing data between different experimental conditions or different organisms, and integrating different kinds of omics data. While data from thousands of single cells can be tricky to analyze, software advances are making it easier. It is expected that more sophisticated and useful analytical frameworks and advanced software will be rapidly developed to handle these challenges. One frustration that accompanies scRNA-seq is a high instance of noise, and subtle changes in amplification and capture efficiency can result in huge differences between cells, in the case of such small amounts of starting RNA material. We must be vigilant against batch effects and introducing variation in samples due to different processing dates or use of different reagent batches.

In conclusion, scRNA-seq is a powerful technique that can enable better understanding of normal and malignant hematopoiesis although some challenges remain to be resolved. The advent of novel technologies, such as the 10X Genomics based GemCode platform and the Chromium single-cell 3′-solution, further drives the application of scRNA-seq. We envision that the wide application of scRNA-seq to study the hematopoietic microenvironment will be very helpful for understanding the complexity of hematopoiesis and will provide insights into the diagnosis and prognosis of patients with hematological diseases.

## Author Contributions

XH wrote this review, PQ and YZ searched some of the literature, SX and XL revised the manuscript, and SX and JC designed the framework. SX and JC are co-corresponding authors.

### Conflict of Interest Statement

The authors declare that the research was conducted in the absence of any commercial or financial relationships that could be construed as a potential conflict of interest.
